# Remarks on the Infantile Fever, and on the Nature of Fever in General

**Published:** 1810-02

**Authors:** 


### *Remarks on the Infantile Fever, and on the Nature of Fever in general.*

THE difficulty of distinguishing the diseases of children was never greater than at the present, owing to the various hypotheses that do but rise to fall, and which serve only to confound the timid and fluctuating mind of the young practitioner. Were medical men to establish theories, by drawing unshaken inferences from facts, instead of accommodating symptoms to any particular hypothesis, which, for the moment, usurps the empire of reason, we should be no longer driven backward and forward on the agitated agitated waves of uncertainty. What must be the state of our knowledge, when in every publication on the diseases of children, the symptoms of hydrocephalus acutus and infantile fever are described as being exactly similar; and when black putrid stools are denominated hydrocephalic?

However some practitioners of respectable authority deny the frequency of hepatic affections in this climate, certain I am, they form no small proportion, as well of the diseases of children as of adults; that mercury will cure them, I am equally certain; but whether or no any other article of the materia medica have like efficacy, I cannot from experience determine. No medicine that I have tried has ever been attended with such happy effects, though the cause of such effects, or the modus operandi of the medicine, seems to be involved in obscurity.

When we are called to a child labouring under what is called the infantile fever, we are told that he has been poorly for some days; we find that he has no appetite; his bowels costive; and perhaps there is nausea, or vomiting. The child, if he be of an age to admit of it, will complain of violent head-ache, thirst, and a burning heat of the skin; he will start during sleep, and pick his nostrils. The eyes look suffused and heavy; and the tongue, on examination, will be covered with fur, independently of that whiteness which seems natural to the tongues of children. How are we to account for such symptoms, and consequently, what practice is to be pursued?

*The violent head-ache, flushed face, picking of the nostrils,* and *starting during sleep,* will, on the first blush of the subject, tell us these are symptoms of hydrocephalus acutus. Having thus discovered what we imagine to be the nature of the disease, we shall begin to deplete with the lancet or leeches, to blister the head, and besides putting our little patient to much unnecessary torture, induce a state of debility, from whence he may never recover; for so important a fluid as the blood cannot be formed without a due and energetic performance of the digestive process; this being put a stop to, the springs of life are dry, the waste of the system goes on, and death ensues from inanition. This description may be said to be too highly coloured; it may be so! yet the leading features of it, I doubt not, have been too often realized in the depletory practice of those who entertain too high an opinion of the lancet.

The next question which naturally occurs, and which is by far the most important, inasmuch as it leads to successful practice, is, how are we to account unequivocally for such symptoms? This calls our attention to organs no less important than the brain, I mean the digestive organs. The bowels are constipated; there is no appetite; there is nausea, &c.; appearances manifestly evincing a derangement of the chylo-poietic viscera; but can this derangement account for the prostration of strength, heat of the surface, and the symptoms affecting the head? I think it can.

If we inquire what kind of evacuation the child has had prior to his having any noticeable complaint, we shall be told that it was black, small in quantity, and of an offensive smell; that, in short, it evinced no appearance from whence to infer the presence of bile. This leads us to the source of the disease. From whatever accidental cause, as cold, improper food, &c. the functions of the liver are either suspended, or the biliary ducts obstructed, the consequence is, that the aliment is incompletely digested; fæcification does not go on; the intestines are torpid from the want of their accustomed stimulus; and hence, the want of colour and retention of the fæces. If then the half-formed fæces remain in the intestines, without the addition of bile, they will, from the heat of the body, be in the most convenient situation for putrefaction; which must inevitably take place. The consequence is, that the more fluid parts of this putrid mass are taken into the circulation by absorption; this depresses the system, and perhaps contributes to produce the other phænomena; for if contagious matter, in the form of miasmata, will produce such direful effects on the animal economy, it is but fair to conclude, that putrid matter in its present form or modification will occasion the symptoms in question. Thus, then, we have accounted for the prostration of strength; "that nausea or vomiting takes it rise from thence needs no proof; there existing such a well known sympathy amongst the digestive organs, owing to their mutual dependance on each other."

Admitting this to be true, I think we are justifiable in attributing to the same cause, the alternations of heat and cold on the surface; for that there is direct sympathy between the skin and digestive organs, numerous proofs might be adduced; at present, however, I shall confine myself to a few familiar examples. It is well known that when a person is about to vomit, this organ is variously affected; at one moment the patient is chilled with a sense of of coldness, (the skin exhibiting the appearance called goose-skin); then again, he complains of intolerable heat. Now he is covered with a cold clammy sweat; then a profuse perspiration emanates from every pore. It is equally certain, that in some peculiarities of constitution, the eating of shell-fish and other species of aliment will be followed by an efflorescence on the skin. But here it may be objected, that in the examples I have brought forward the stomach was solely affected; and that as it is an universal sympathizer, it was sufficient to produce such symptoms; this I grant: but if we allow that the primary disease existed in the liver, and that in consequence of such disease, the stomach and bowels were secondarily affected, immediately the objection will vanish, and we shall be no longer at a loss to account for the phænomena in question. Now, with respect to the flushing of the face; those who argue for cerebral inflammation being the proximate cause of fever, will assert, that *this* proves a determination of blood to the head, and from thence they infer an actual inflammation of the brain. The fallacy of this can be easily detected; for as we have just shewn the sympathy between the skin and chylo-poietic viscera, and have proved the cause of the alternations of heat and cold on the surface in general; can we wonder that it here evinces a florid hue, where it is so plentifully supplied with vessels? To say that this was a proof of cerebral inflammation would be tantamount to saying that a transient blush denoted an incipient phrenitis.

The next set of symptoms that need explanation, and which those of the opposite opinion deem insurmountable, I shall proceed to investigate: I allude to the head-ach, starting during sleep, and picking of the nostrils. That pain of the head, starting during sleep, or convulsions, may arise from an affection of the chylo-poietic viscera, can be easily accounted for on the grounds of sympathy. Now the brain being the common sensorium, must sympathize with the rest of the system; that it does so, I need but call your attention to the head-ach and delirium that attend a symptomatic fever, arising from any considerable local injury. If then an injury done to a limb will give rise to such strange affections of the brain and nervous system, a fortiori, the effect must be greater when such important organs as the abdominal viscera being deranged, are the exciting cause. This being granted, we have only to prove a positive affection of the one, and no manifest disease of the other, to establish the point; and here dissection section is decidedly in our favour, for we always find after death an inflammation of the mucous membranes, liver, spleen, or any other of the abdominal viscera; while the brain presents an appearance perfectly natural, there existing no sensible change of structure. Dr. Fordyce, and many other physicians of equal eminence, whose extensive opportunities for observation render their testimony unimpeachable, never found any disorganization of the brain in patients who died of typhus fever, while the digestive or pneumonic organs always evinced marks of disease.

As to the child's picking his nostrils, this cannot argue an inflammation of the brain, for as the Sniderian membrane is but a continuation of the mucous membrane lining the alimentary canal, (though it differs somewhat in structure) it is but rational to suppose, that when one part is affected, the other will be affected sympathetically; nor is evidence wanting on this point, for who will doubt the sympathetic consent of the membranes of the nostrils, fauces, ductus nasalis, and eye, when his fauces are irritated by too large a proportion of any stimulating condiment, as mustard, &c.? Admitting then a partial, we cannot hesitate to allow a general sympathy; and the moment this is granted, the fact in question is established; and we at once trace back the symptom to its original source.

Having thus, I trust, satisfactorily proved the proximate cause of the disease, or, in other words, the nature of the disease itself, the indications of cure are obvious, viz. to evacuate the intestines of their putrid contents, and thus relieve the symptoms, at the same time, by inducing a due secretion of bile to prevent their recurrence; that mercury will have these effects when administered early, at least before the absorption of contagious matter has been so great as to render the effects of medicine futile, I shall endeavour to prove by the recital of a few cases.

If we admit this Theory to be true, we cannot wonder that bark, wine, acids, &c. cure typhus; at least, support the sinking powers of the system till the poison has spent its force.

CASE I, Nov. 25, 1809. Master C———, æt. 4, having been unwell for some days, complained about midnight of a violent head-ach, thirst, and a burning heat of the surface. I saw him the day following, in the evening; I found him with a flushed face; eyes dull and heavy; head-ach, and costiveness, having had no stool for some days. He has no appetite, and is unable to walk about from prosprostration of strength. His tongue is furred, and pulse quick; but as the circulation goes on with great rapidity in children, I did not attend to the latter symptom.

Mitte hydrargyri submuriatis, gr. v. triturated with an equal quantity of sugar; to be taken immediately: and after two hours, a little of the sulphate of magnesia dissolved in infusion of senna.

I ordered the patient to be laid in a recumbent posture, and the head kept wet with vinegar and water, to cause an evaporation from the surface, fearing from the accounts that are set down in books, an incipient hydrocephalus. The mercury produced evacuations upwards and downwards, the fæces were black, and of an intolerably offensive smell. The child expressed himself as being nearly well, and the eyes seemed to have recovered their wonted vivacity.

Nov. 27. Our patient has had no stool, and no return of appetite. Rep. pulvis calomelanos statim. sumendus. This was again productive of the most happy effects; he had several evacuations which gradually returned to their natural colour; and the day following, the child was completely recovered.

Case II. The sister of the above child was soon after affected with the same set of symptoms, and as speedily recovered by the use of calomel.

Case III. Nov. 28. Mr. M's child, aged 3, of an extremely delicate habit, has been indisposed for three or four days, in consequence of having, as they supposed, caught a severe cold. She has violent fits of screaming, starts during sleep, and is incessantly picking her nose. She seems to refer her complaint chiefly to her head; her face is alternately flushed, and pale; the eyes look dull and heavy; and the pupils seem considerably dilated. She has an incessant cough, and breathes laboriously; her bowels have been some days constipated; and she refuses food. Still haunted with the fears of hydrocephalus, I gave directions about wetting the head, as in the preceding cases, previously proposing the application of leeches, and a blister, which, happily for the patient, the parents would not accede to.

Mitte hydrargyri submuriatis, gr. iv. statim sum.; and after two hours, a little of the solution, which indeed, could not be administered.

Nov. 29. The calomel produced four copious, black, and offensive stools. The child seems much relieved, though she still refuses her food, and is incapable of motion; lying constantly on her back. Rep. pulv. calomelanos.

30. The

30. The stools still evince no bile, but she was able to walk about, and has taken a little nourishment. Rep. pulv. calomelanos.

31. The stools now resumed their natural colour; the cough and difficuly of breathing disappeared; in short, she was *completely* recovered by good diet and tonic medicines.

CASE IV. Dec. 10, 1809. I was requested to visit Miss S. æt. 4, who is naturally of a robust constitution, but has been some days indisposed. She complains of a pain in her head, and is unable to move her lower extremities. Her face is flushed; eyes heavy, and pupils considerably dilated. She has had no sleep for some nights; refuses food; and has had no evacuation since she began to be ill. Mitte hydrargyri submuriatis, gr. viij. statim sum.

11. The calomel produced its usual effects; she has had an indifferent night, but is apparently better. Rep. pulv. calom.

12. The stools evinced bile; she could now walk; her appetite returned; and a little of the decoction of bark completely restored her.

I am, &c.

JUNIUS.

*January* 11, 1810.

